# Scheduling of nitrogen fertilizer topdressing during panicle differentiation to improve grain yield of rice with a long growth duration

**DOI:** 10.1038/s41598-020-71983-y

**Published:** 2020-09-16

**Authors:** Yang Liu, Xinguang Zhu, Xiaoe He, Chao Li, Tiangen Chang, Shuoqi Chang, Haiqing Zhang, Yuzhu Zhang

**Affiliations:** 1 Southern Regional Collaborative Innovation Center for Grain and Oil Crops (CICGO), Hunan Agricultural University, Changsha, 410128 China; 2 Hunan Rice Research Institute, Hunan Academy of Agricultural Sciences, Changsha, 410125 China; 3 State Key Laboratory of Hybrid Rice, Hunan Hybrid Rice Research Center, Hunan Academy of Agricultural Sciences, Changsha, 410125 China; 4 National Key Laboratory of Plant Molecular Genetics, CAS Center for Excellence in Molecular Plant Sciences, Shanghai Institute of Plant Physiology and Ecology, Chinese Academy of Sciences, Shanghai, 200031 China; 5 Hunan Soil and Fertilizer Research Institute, Hunan Academy of Agricultural Sciences, Changsha, 410125 China

**Keywords:** Fertilization, Field trials

## Abstract

Topdressing at panicle differentiation (PF) according to soil fertility and regularity of rice nutrient absorption is an important agronomic practice used in cultivation of rice cultivars with a long growth duration. We studied the impacts of timing of nitrogen fertilizer application during PF on photosynthesis and yield-related agronomic traits in ‘Y-Liang-You 900’ and ‘Y-Liang-You 6’, which are representative rice cultivars with a long growth duration. Data for two years showed that timing of topdressing application during PF affected panicles per unit area, percentage grain set, spikelets per panicle, and leaf photosynthetic traits during the grain-filling period. Topdressing at the initial stage of flag-leaf extension resulted in higher grain yield (typically by 10.55–19.95%) than in plants without topdressing. Grain yield was significantly correlated with flag leaf photosynthetic rate and leaf SPAD value (*r* = 0.5640 and *r* = 0.5589, respectively; *p* < 0.01) at an advanced grain-filling stage (30 days after heading). Surprisingly, grain yield was not correlated with carbohydrate remobilization from the stem and sheath. For rice cultivars with a long growth duration, nitrogen-fertilizer topdressing must be applied at the initial stage of flag-leaf extension to delay leaf senescence during the grain-filling stage and realize the enhanced yield potential.

## Introduction

Rice (*Oryza sativa* L.) is a staple food for more than one-third of the global population^1,2^. Rice grain yield is required to increase by more than 1% annually to meet the expected increase in demand as a result of continued population growth and decrease in available paddy area^3–5^. Breeding of high-yielding cultivars and determination of optimal agronomic practices for different cultivars are required to realize the required improvement in rice yield^6^. Actual grain yield is the product of the harvest index (HI) and biomass. The HI of many elite rice cultivars has already attained an extremely high value (above 0.5), thus further improvement of the yield potential is reliant on an increase in biomass^7^. This realization is already reflected in current rice breeding programs, in which genotypes with a long growth duration, characterized by a longer grain-filling period, are selected for greater biomass production and correspondingly higher grain yield at harvest, as exemplified by ‘Y-Liang-You 900’^8–10^. ‘Y-Liang-You 900’ differs from many rice cultivars, which typically have a growth duration of 110–120 days, in having a growth duration of more than 140 days^11^. This comparatively long growth duration necessitates development of novel field practices to realize the yield potential of such high-yielding rice lines in the field.

A number of field practices are now routinely used in modern rice cultivation, including promotion of germination in a seedbed, organic fertilizer application before transplanting, water management during the tillering stage, topdressing during the young panicle differentiation stage, and alternate wetting and drying during the grain-filling stage^10,12^. Among these practices, different topdressing schedules can differentially influence photosynthetic efficiency^13^ and panicle differentiation, thereby resulting in differences in panicle number per plant and number of seeds per panicle^14–16^, and the functional longevity of leaves during the grain-filling stage^17–19^.

The process of young panicle differentiation of rice can be divided into eight stages^20^. The effects of fertilizer topdressing on leaf photosynthesis and panicle development differ, depending on the stage of topdressing application, in rice cultivars with a relatively short growth duration^21^. However, little information is available on the effects of topdressing techniques during panicle development on rice cultivars with a long growth duration. In this study we investigated the effects of different topdressing schedules during panicle development on photosynthetic traits at advanced developmental stages and grain yield of two representative rice cultivars with a long growth duration. The results provide information to achieve high-yield cultivation of rice cultivars with a long growth duration.

## Materials and methods

### Field management

Experiments were conducted at the experimental station of the Hunan Rice Research Institute, Changsha, China (28°11′59″ N, 113°04′35″ E) in 2016 and 2017. The field soil properties analyzed before transplanting were as follows: organic matter 22.4 g kg^−1^, total N 2.9 g kg^−1^, available P 28.1 g kg^−1^, and available K 186.7 g kg^−1^. Two *indica* hybrid rice cultivars, namely ‘Y-Liang-You 900’ (YLY900; raised from the cross of a female parental line Y58S and a male parental line R900) and ‘Y-Liang-You 6’ (YLY6; derived from the cross of a female parental line Y58S and a male parental line Wanghui006), were used. The growth duration of these two cultivars is about 140 days^10^.

A split-plot design was applied with nitrogen fertilizer treatments as the main plots and the rice cultivars as the subplot. Each experiment was replicated three times and the subplot area was 30 m^2^. Seven treatments were applied consisting of five topdressing treatments at different stages of panicle development, namely exsertion of the fifth leaf (PF-1), fourth leaf (PF-2), third leaf (PF-3), second leaf (PF-4), and flag leaf (PF-5) counted from the shoot tip (collectively PFs), together with a no topdressing treatment (NPF) and a no fertilizer treatment (NF). The leaf age of 15 plants was marked in each plot to determine the timing of topdressing application during panicle development. The timing of topdressing was determined on the basis of exsertion of the *n*th leaf from the shoot tip and the length of young panicles, following Yoshida^39^ and Chen et al.^20^ The timing and amount of nitrogen fertilizer applied for the seven treatments is summarized in Table 1. Potassium was applied at two growth stages, i.e., before transplanting as a basal dressing and as a topdressing at panicle development stage at the rate of 150 kg K_2_O ha^−1^. All phosphorus (150 kg P_2_O_5_ ha^−1^) was applied as basal fertilizer 1 day before transplanting.

**Table 1:** Timing and rate (kg N ha^−1^) of topdressing with nitrogen fertilizer for each treatment.

Developmental stage		1 day before trans-planting	7 days after trans-planting	Elongation stage	Neck node differentiation	Primary branch differentiation	Middle differentiation of spikelet	Pollen mother cell differentiation
Exsertion of *n*th leaf counted from the shoot tip		–	–	Fifth	Fourth	Third	second	Flag leaf
Date	2016	29th May	6th June	9th July	15th July	22nd July	30th July	7th August
	2017	20th May	28th May	30th June	6th July	13th July	21st July	29st July
Treat	PF-1	120	60	90	0	0	0	0
	PF-2	120	60	0	90	0	0	0
	PF-3	120	60	0	0	90	0	0
	PF-4	120	60	0	0	0	90	0
	PF-5	120	60	0	0	0	0	90
	NPF	120	60	0	0	0	0	0
	NF	0	0	0	0	0	0	0

Germinated seeds were sown in a nursery bed on 8 May 2016 and 29 April 2017. Seedlings were transplanted 22 days after sowing with two seedlings per hill at a density of 20 cm × 30 cm. Except for mid-growth-season drainage, the field was continuously flooded with 3–5 cm water until 1 week before the final harvest. Insects, weeds, and diseases were controlled following local agronomic practices.

### Experimental sampling and measurements

Six hills were sampled in each plot at the heading stage and at maturity with three replications. The sampled plants were separated into leaves, stems, and panicles. The dry weight of vegetative organs (DWVO) was measured after oven-drying at 70 °C to constant weight. Six plants sampled at maturity were threshed after the panicle number was counted. Unfilled spikelets were separated from filled spikelets using tap water, then filled and unfilled grains were counted to determine the spikelet number per panicle and percentage grain set. The filled grains were weighed to determine the 1,000-grain weight. Grain yield was determined from an 8 m^2^ area in the center of each plot. The grain yield was adjusted with a moisture content of 14%.

Net photosynthetic rate (*P*_n_) in the medial part of the flag leaf was measured between 09:00 and 11:00 with a portable photosynthesis system (LI-6400, LI-COR, Lincoln, NE, USA) at the heading stage, 15 days after heading, and 30 days after heading. The photosynthetic photon flux density was set to 1,000 μmol m^−2^ s^−1^, and the leaf temperature and CO_2_ concentration during measurement were 28 °C and 380 ppm, respectively. Chlorophyll content of flag leaves (at the center of the leaf), as represented by the SPAD value, was measured using a SPAD-502 chlorophyll meter (Minolta Camera Co., Ltd, Osaka, Japan).

The remobilization rate of reserves in vegetative organs (RRVO) was defined as the ratio between the remobilized reserved organic matter from vegetative organs and the total DWVO at heading. The contribution of reserved organic matter in vegetative organs to grain yield (CRVO) was defined as the apparent contribution of preheading reserves to grain yield. Specifically, RRVO and CRVO were calculated using the following formulas:

$$ \begin{gathered} {\text{RRVO }} = \, \left( {{\text{DWVO at heading stage }}{-}{\text{ DWVO at mature stage}}} \right)/{\text{DWVO at heading stage}} \hfill \\ {\text{CRVO }} = \, \left( {{\text{DWVO at heading stage }}{-}{\text{ DWVO at mature stage}}} \right)/{\text{Grain yield}}. \hfill \\ \end{gathered} $$

### Statistical analysis

Each treatment was assigned with three replications. All experimental data presented are the average of the three replicates. Analysis of variance (ANOVA) was conducted using SAS release 6·12 (SAS/ETS 1993) under the split-plot design to detect significant differences among treatments. For the experimental indicators ‘spikelet’ and ‘panicle number’, observations were arcsine transformed for adherence to the assumptions of additivity between experimental effects and errors, normal distribution of the data, and homogeneity of error variances before performing ANOVA. A *post-hoc* Tukey's honestly significant difference test was used as a parallel test (or named as multiple comparisons) in ANOVA. The significance level for the *F*-test or parallel test was set to 0.05. The standard error (SD) for each mean is presented in the graphs as error bars.

## Results

### Grain yield and yield-related agronomic traits

**Figure 1:**
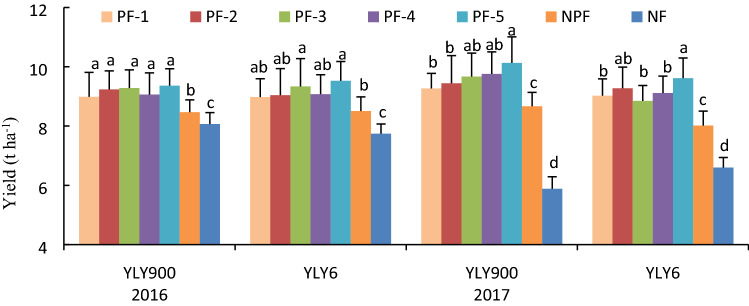
Grain yield of the rice cultivars ‘YLY900’ and ‘YLY6’ under the nitrogen fertilizer treatments. Each value is the mean of three replicates. Bars with different lower-case letters indicate significant differences for yield at p < 0.05 among seven panicle fertilizer treatments in 2016 or 2017, respectively.

The grain yields in the PF treatments of YLY900 were 8.98–9.36 t ha^−1^ and 9.27–10.13 t ha^−1^ in 2016 and 2017, respectively, and those of YLY6 were 8.81–9.53 t ha^−1^ and 9.02–9.62 t ha^−1^, respectively (Fig. 1). The grain yields in the PFs were significantly higher than those of NPF and NF for both cultivars in the two years, with increases of 5.78–11.23% in 2016 and 9.67–18.36% in 2017 compared with yields under NPF, and increases of 13.53–19.39% in 2016 and 46.59–58.20% in 2017 compared with those under NF. The grain yields also differed among the five PFs. The PF5 treatment consistently resulted in the highest grain yield across cultivars and years (Fig. 1).

Panicle number on a unit area basis and percentage grain set differed significantly among treatments, whereas the differences in spikelet number per panicle and 1,000-grain weight were relatively small or inconsistent across cultivars and/or years (Table 2). The panicle number of PFs ranged from 212.28 × 10^4^ to 237.24 × 10^4^ ha^−1^, which was higher than that of the NPF and NF treatments by approximately 9% and 42%, respectively. However, with regard to the average panicle number, the rank order of the PFs for each cultivar was inconsistent, i.e., the order was PF-2 > PF-3 > PF-5 > PF-4 > PF-1 for YLY900, and PF-1 > PF-2 > PF-3 > PF-5 > PF-4 for YLY6 (Table 2). For spikelet number per panicle across PFs, the observed order was PF-5 > PF-4 > PF-3 > PF-2 > PF-1 for YLY900, and PF-4 > PF-5 > PF-1 > PF-2 > PF-3 for YLY6. Specifically, PF-4 and PF-5 showed the highest spikelet number per panicle, which was significantly higher than that of NPF (Table 2), whereas the differences between PF-1, PF-2, PF-3, and NPF were inconsistent across cultivars and/or years. Regarding percentage grain set, NF resulted in the highest grain set of approximately 90%, whereas PF-5 resulted in percentage grain set of 83.59% and 88.94% for YLY900, and 83.29% and 85.87% for YLY6 in 2016 and 2017, respectively, which were higher than those observed for NPF and the other PF treatments (Table 2).

**Table 2:** Grain yield and yield-related agronomic traits of the rice cultivars ‘YLY 900’ and ‘YLY 6’ under the nitrogen fertilizer treatments.

	YLY900				YLY6			
	Panicles ha^−1^ (× 10^4^)	Spikelets panicle^−1^	Grain setting rate (%)	1,000-grains weight (g)	Panicles ha-^1^ (× 10^4^)	Spikelets panicle^−1^	Grain setting rate (%)	1,000-grains weight (g)
**2016**								
PF-1	194.6 ± 7.3ab	250.5 ± 16.2ab	81.4 ± 2.1ab	23.3 ± 0.4a	209.5 ± 11.4b	204.0 ± 9.6a	83.8 ± 2.2c	25.4 ± 0.6a
PF-2	209.4 ± 11.5a	259.9 ± 11.7a	74.0 ± 0.8c	23.3 ± 0.6a	239.2 ± 17.2a	196.3 ± 13.1ab	77.7 ± 0.8d	25.1 ± 0.3a
PF-3	204.2 ± 6.9a	260.2 ± 13.1a	82.7 ± 1.6a	22.6 ± 0.3ab	230.5 ± 14.6a	198.8 ± 7.5a	85.0 ± 1.3bc	25.5 ± 0.3a
PF-4	187.8 ± 9.0b	267.2 ± 17.2a	83.2 ± 2.0a	22.3 ± 0.7b	221.1 ± 9.7ab	201.6 ± 14.4a	87.1 ± 1.0ab	25.0 ± 0.5a
PF-5	195.7 ± 13.1ab	274.5 ± 11.0a	83.5 ± 1.2a	22.2 ± 0.5b	228.6 ± 15.5a	195.7 ± 10.0ab	88.9 ± 1.5a	25.1 ± 0.7a
NPF	189.3 ± 7.4b	261.0 ± 11.4a	79.0 ± 0.9b	22.7 ± 0.3ab	212.7 ± 14.0b	181.4 ± 11.3b	86.1 ± 0.9b	25.6 ± 0.4a
NF	171.0 ± 6.8c	244.6 ± 10.9b	85.8 ± 1.5a	23.1 ± 0.3a	199.5 ± 10.6c	179.7 ± 8.8b	89.8 ± 1.1a	25.4 ± 0.3a
**2017**								
PF-1	264.1 ± 17.4a	226.8 ± 12.6b	74.1 ± 0.9d	23.8 ± 0.5a	280.7 ± 20.3a	193.0 ± 8.6a	76.6 ± 1.3d	27.0 ± 0.7b
PF-2	232.2 ± 14.7b	234.9 ± 15.4ab	77.4 ± 1.1c	24.7 ± 0.4a	255.3 ± 17.7b	173.3 ± 10.1bc	83.1 ± 0.9bc	28.2 ± 0.5a
PF-3	229.6 ± 9.8b	236.2 ± 11.3ab	78.9 ± 1.5bc	24.9 ± 0.7a	251.3 ± 12.8b	166.6 ± 7.9c	77.8 ± 0.7d	28.2 ± 1.0a
PF-4	224.5 ± 11.5b	255.8 ± 11.0a	76.4 ± 0.5c	24.7 ± 0.3a	236.0 ± 15.4c	181.4 ± 8.0ab	82.8 ± 1.4bc	28.4 ± 0.7a
PF-5	220.7 ± 15.3b	246.2 ± 16.1a	83.2 ± 1.7a	24.6 ± 0.8a	242.6 ± 11.9bc	176.6 ± 9.2bc	85.8 ± 0.5b	28.8 ± 1.2a
NPF	227.1 ± 12.2b	222.4 ± 11.8b	79.6 ± 1.0b	24.3 ± 0.6ab	238.8 ± 13.3c	165.6 ± 7.1c	81.5 ± 1.0c	28.0 ± 0.8ab
NF	131.4 ± 10.0c	243.0 ± 13.7a	81.2 ± 1.2ab	23.6 ± 0.4b	164.6 ± 11.7d	173.6 ± 10.3bc	88.6 ± 1.4a	27.0 ± 0.8b

Each value is the mean with standard error (± SD), n = 6. Different lower-case letters indicate significant differences for Panicles ha^−1^, Spikelets panicle^−1^, Grain setting rate or 1,000-grains weight at p < 0.05 among seven panicle fertilizer treatments in 2016 or 2017, respectively.

### Net photosynthetic rate and SPAD value of flag leaves

**Figure 2:**
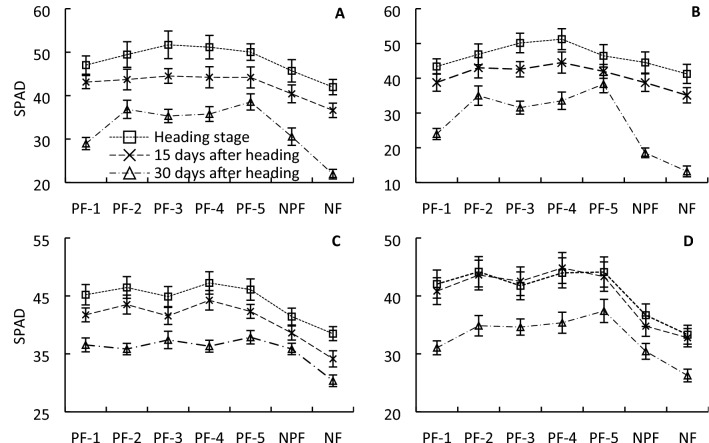
Flag leaf SPAD value of ‘YLY 900’ (**A**,**C**) and ‘YLY 6’ (**B**,**D**) in 2016 (**A**,**B**) and 2017 (**C**,**D**) under the nitrogen fertilizer treatments. Each value is the mean of three replicates.

**Figure 3:**
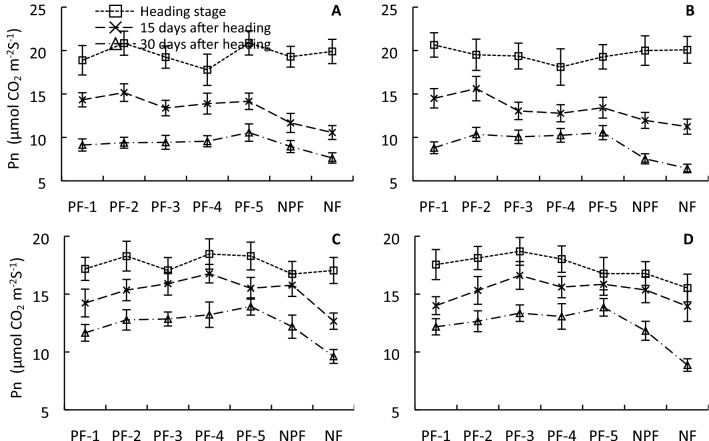
Net photosynthetic rate (Pn) of ‘YLY 900’ (**A**,**C**) and ‘YLY 6’ (**B**,**D**) in 2016 (**A**,**B**) and 2017 (**C**,**D**) under the nitrogen fertilizer treatments.

All PFs increased the SPAD value of the flag leaf at the heading stage, 15 days after heading, and 30 days after heading. PF-4 resulted in the highest SPAD value at the heading stage and 15 days after heading, whereas plants under PF-5 showed a remarkably high SPAD value of 39.79 at 30 days after heading, which was 6.93–73.43% higher than that of all other treatments (Fig. 2). Interestingly, PFs did not significantly change the flag leaf *P*_n_ at the heading stage, which was around 18 μmol CO_2_ m^−2^ s^−1^ for all treatments; however, the PFs significantly increased the *P*_n_ of the flag leaf at 15 and 30 days after heading compared with that of NPF and NF plants. Specifically, plants under PF-2 showed the highest leaf *P*_n_ of 19.19 μmol CO_2_ m^−2^ s^−1^ at the heading stage, whereas plants under PF-5 showed the highest leaf *P*_n_ of 12.22 μmol CO_2_ m^−2^ s^−1^ at 30 days after heading, which was 6.20%–20.01% higher than that of the other PFs and 27.70% higher than that of NPF (Fig. 3).

### Remobilization efficiency of reserves in vegetative organs

The NF treatment showed the lowest DWVO at the heading and harvest stages for both cultivars in both years, whereas PF-2 and PF-1 showed the highest DWVO at the heading and harvest stages for both cultivars in 2016 and 2017, respectively (Table 3). Notably, PF-5 resulted in a high DWVO at the heading stage for both cultivars in both years. NF led to the highest RRVO and CRVO values among all treatments, i.e., values of 18.96–28.58% and 23.47–33.28% across the two cultivars and growth years. Remarkably, although no consistent trends were observed for changes in RRVO and CRVO in the other PFs compared with those of NPF, PF-5 resulted in high RRVO and CRVO values in the two cultivars and years, which indicated that PF-5 showed a consistent effect in promoting reserves remobilization (Table 3).

**Table 3:** Dry matter accumulation in vegetative organs and transport to reproductive organs under the nitrogen fertilizer treatments.

	YLY900				YLY6			
	DWVO at heading stage (t ha^−1^)	DWVO at mature stage (t ha^−1^)	RRVO (%)	CRVO (%)	DWVO at heading stage (t ha^−1^)	DWVO at mature stage (t ha^−1^)	RRVO (%)	CRVO (%)
**2016**								
PF-1	10.2 ± 0.3b	8.3 ± 0.5b	17.9 ± 1.8c	20.4 ± 1.0c	10.5 ± 0.6a	9.8 ± 0.2ab	6.5 ± 0.5d	7.6 ± 0.3d
PF-2	11.4 ± 0.6a	9.2 ± 0.3a	19.1 ± 1.3b	23.6 ± 1.4b	11.0 ± 0.3a	10.2 ± 0.5a	7.1 ± 0.2d	8.7 ± 0.3d
PF-3	10.1 ± 0.2b	8.9 ± 0.3ab	11.8 ± 0.8d	13.0 ± 0.9e	10.5 ± 0.7a	9.8 ± 0.4ab	6.7 ± 0.3d	7.6 ± 0.5d
PF-4	9.8 ± 0.4b	8.3 ± 0.2b	15.2 ± 1.1c	16.6 ± 0.7d	9.9 ± 0.4bc	8.8 ± 0.5c	11.7 ± 0.7b	12.9 ± 0.7bc
PF-5	11.0 ± 0.7a	8.5 ± 0.6b	22.7 ± 1.5a	26.8 ± 1.3ab	10.6 ± 0.6a	9.3 ± 0.7b	12.7 ± 0.5b	14.2 ± 1.0b
NPF	10.6 ± 0.2ab	8.7 ± 0.5ab	18.2 ± 0.9bc	21.9 ± 1.3bc	10.1 ± 0.9b	9.1 ± 0.4bc	10.2 ± 0.7c	11.7 ± 0.8c
NF	9.9 ± 0.3b	7.6 ± 0.6c	23.6 ± 1.7a	29.2 ± 1.6a	9.6 ± 0.5c	7.8 ± 0.3d	18.9 ± 0.9a	23.6 ± 1.6a
**2017**								
PF-1	11.6 ± 0.4a	8.7 ± 0.5a	25.0 ± 1.4b	30.3 ± 2.4ab	10.5 ± 0.7a	8.4 ± 0.5a	19.5 ± 1.2b	20.0 ± 1.2b
PF-2	10.0 ± 0.6bc	8.1 ± 0.6ab	19.1 ± 1.8d	20.4 ± 1.8e	8.6 ± 0.3c	7.6 ± 0.2b	11.1 ± 0.8c	10.3 ± 0.7d
PF-3	10.4 ± 0.7b	7.8 ± 0.3b	24.8 ± 1.2b	26.7 ± 2.0c	9.1 ± 0.4bc	8.0 ± 0.4ab	12.1 ± 0.6c	13.3 ± 0.6c
PF-4	8.9 ± 0.4c	6.7 ± 0.2c	24.4 ± 1.5bc	22.5 ± 1.7d	8.3 ± 0.2c	7.6 ± 0.6b	8.3 ± 0.5d	7.6 ± 0.5e
PF-5	10.9 ± 0.4ab	8.0 ± 0.5ab	26.4 ± 1.9ab	28.5 ± 2.1bc	10.1 ± 0.7ab	8.3 ± 0.5a	17.5 ± 1.0b	18.3 ± 0.9b
NPF	9.7 ± 0.6c	7.4 ± 0.4b	23.9 ± 0.9c	26.9 ± 2.2c	7.8 ± 0.4d	6.3 ± 0.4c	19.2 ± 1.3b	18.8 ± 1.0b
NF	6.8 ± 0.3d	4.8 ± 0.3d	28.5 ± 1.7a	33.2 ± 1.7a	6.5 ± 0.3e	5.0 ± 0.4d	23.5 ± 1.6a	23.4 ± 1.7a

Each value is the mean with standard error (± SD), n = 6. Different lower-case letters indicate significant differences for DWVO at heading stage, DWVO at mature stage, RRVO or CRVO at p < 0.05 among seven panicle fertilizer treatments in 2016 or 2017, respectively.

## Discussion

### Topdressing at the initial stage of flag-leaf extension can significantly increase grain yield in YLY900 and YLY6 compared with no topdressing

The amount and timing of fertilizer applied is critical to realize the yield potential of rice in the field. Extensive research has been conducted to identify the optimal nitrogen application scheduling for earlier-maturing rice cultivars with a relatively lower yield potential and shorter growing season. For example, under a nitrogen application rate exceeding 200 kg N ha^−1^, the rice grain yield is not further increased and yield might even decline with a higher nitrogen application rate^22–24^. In the present study, we observed that the optimal fertilizer quantity required for YLY900 and YLY6 to attain the greatest gain in yield was considerably higher than that reported for earlier-maturing cultivars. Specifically, grain yield can be greatly increased from a total nitrogen application of 180 kg N ha^−1^, i.e., 120 kg N ha^−1^ as basal fertilizer and 60 kg N ha^−1^ at the tillering stage, to a rate of 270 kg N ha^−1^, i.e., with an additional 90 kg N ha^−1^ applied as topdressing compared with the former nitrogen application regime (Fig. 1). This finding shows that with the increase in rice yield potential in YLY900 and YLY6, the topdressing quantity needs to be increased to meet the nitrogen demand of these cultivars^10^. Notably, the yield improvements from topdressing at different stages of young panicle development differed, ranging from 5.53 to 19.95% compared with the NF treatment. The present results showed that topdressing at the initial stage of flag-leaf extension resulted in the greatest yield improvement for the cultivars YLY900 and YLY6, which show a long growth duration (Fig. 1). Shortening the basal internodes length and increasing the stem wall thickness are the main strategies to improve the lodging resistance of rice^35^. The two rice cultivars selected for the present experiment were characterized by short basal internodes and thick stem walls, which are beneficial for enhanced lodging resistance. Therefore, no lodging was observed in this experiment under the treatments with increased rates of nitrogen fertilizer application.

### Mechanisms of differential responses of rice yield to topdressing at different stages of young panicle development

Young panicle development of rice is a complex developmental process, classifiable into eight stages, with each stage showing drastically different developmental events. Topdressing at different stages of young panicle development is expected to have different impacts on rice yield. The optimal amount of nitrogen fertilizer applied at the initiation of young panicle differentiation significantly increases spikelet number per panicle, percentage grain set, 1,000-grain weight, and grain yield^25^. Nitrogen utilization efficiency and grain yield are increased by fertilizer application on the eighth day after rewatering^14^. However, to date, the effects on grain yield and yield-related traits of topdressing at different stages of young panicle differentiation have not been systematically studied.

In the present experiment we systematically examined the effects of nitrogen fertilizer applied at different stages of young panicle development on grain yield and yield formation processes. Topdressing at early stages of young panicle development (PF-1, PF-2, and PF-3) caused the panicle number to increase dramatically but the percentage grain set was decreased by as much as 9.77% (Table 2), resulting in a slight or moderate increase in grain yield (Fig. 1). Topdressing at a late stage of young panicle differentiation (PF-5) did not significantly change the panicle number per plant compared with that of NPF, but the spikelet number per panicle and percentage grain set were increased by about 9.12% and 4.67%, respectively, resulting in a significant increase in grain yield (Table 2). These results were consistent with those of Zhang et al.^26^, who reported that applying nitrogen fertilizer coincident with spikelet differentiation and heading increases activities of sucrose synthase and adenosine diphosphoglucose pyrophosphorylase in the inferior spikelets.

The impact of topdressing at advanced stages of young panicle differentiation on grain yield might be associated with enhanced canopy photosynthetic CO_2_ uptake during grain filling. SPAD values reflect the chlorophyll and nitrogen content in leaves, and have been used as an indicator for leaf nitrogen status and leaf photosynthetic capacity^27,28^. The present study showed that topdressing significantly enhanced the SPAD value of leaves during grain filling, especially at advanced grain-filling stages, e.g., 30 days after heading. In particular, in response to topdressing at the initial stage of flag-leaf extension, the SPAD value was 37.92% and 73.43% higher than that of the NPF and NF treatments, respectively, and 32.03%, 6.93%, 14.59%, and 12.87% higher than that of topdressing at the initial stage of fifth-leaf, fourth-leaf, third-leaf, and second-leaf extension, respectively (Fig. 2). In addition to SPAD values, we also directly examined the impact of topdressing on photosynthetic rate. Compared with the NPF treatment, the *P*_n_ at 15 and 30 days after heading was enhanced by as much as 12.22% and 23.84%, respectively. Notably, topdressing at the initial stage of flag-leaf extension resulted in the highest flag leaf *P*_n_ values compared with all other treatments at 30 days after heading for both cultivars across the two years (Fig. 3). The SPAD value of flag leaves was significantly positively correlated with the photosynthetic rate (*r* = 0.5742*, *r* = 0.4380*, and *r* = 0.6928** at the heading stage, 15 days after heading, and 30 days after heading, respectively; * *p* < 0.05, ** *p* < 0.01). These results are in agreement with those of Ma et al., who reported that the SPAD value and chlorophyll content of rice canopy leaves, leaf nitrogen content, and net photosynthetic rate are significantly correlated^29^. The present results indicated that application of nitrogen fertilizer at an appropriate developmental time point, in particular at the initial stage of flag-leaf extension, can significantly improve physiological activity of flag leaves in the middle and advanced grain-filling stages. If the topdressing was not applied, the yield declined by as much as 16.63% (Fig. 1). Therefore, the increase in leaf physiological activity at an advanced grain-filling stage through appropriate topdressing is critical to realize the high-yield potential of rice, in particular in rice cultivars with a long growth duration.

### Post-heading canopy photosynthesis is the dominant source of carbon for grain filling in YLY900 and YLY6

**Figure 4:**
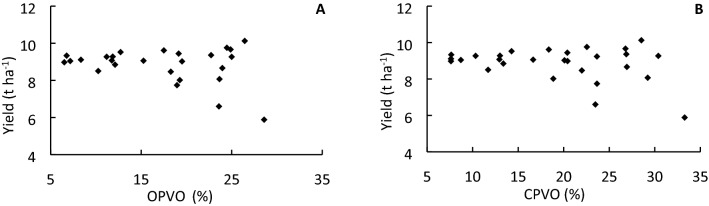
Relationship between grain yield and remobilization rate of reserves in vegetative organs (RRVO; **A**) and contribution of reserved organic matter in vegetative organs to grain yield (CRVO; **B**) in the 2 years, two cultivars, and seven treatments.

**Figure 5:**
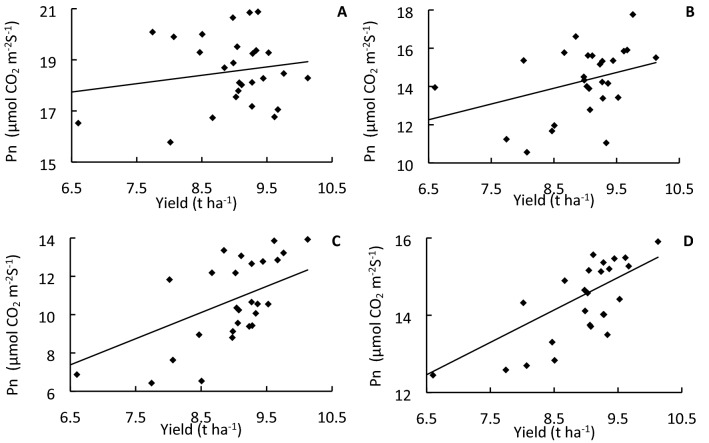
Correlation analysis between grain yield and net photosynthetic rate (Pn) in the two years, two cultivars, and seven treatments. (**A**) Heading stage; (**B**) after heading 15 days; (**C**) after heading 30 days; (**D**) mean of three stages. * and ** indicates significant correlation between yield and *P*n at P < 0.05 and 0.01, respectively.

**Figure 6:**
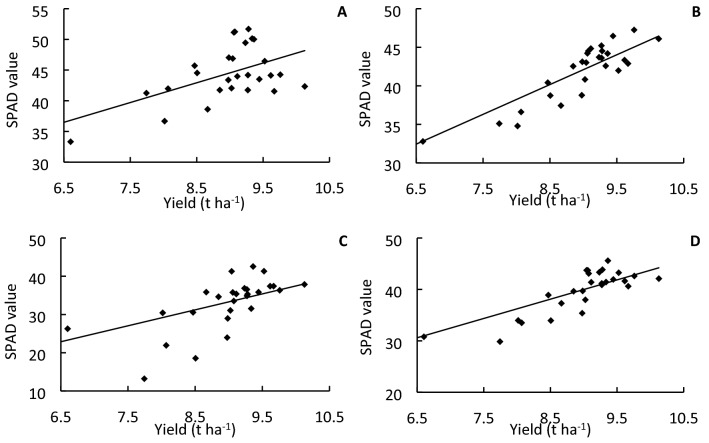
Correlation analysis between grain yield and leaf SPAD value in the two years, two cultivars, and seven treatments. (**A**) Heading stage; (**B**) after heading 15 days; (**C**) after heading 30 days; (**D**) mean of three stages. * and ** indicates significant correlation between yield and SPAD at P < 0.05 and 0.01, respectively.

Postheading photosynthetic CO_2_ uptake and remobilization of preheading carbon reserves contribute to grain yield formation^30,31^. Preheading organic carbon storage in the stem and sheath can potentially contribute to as much as 30% of the grain yield in early-maturing rice cultivars^32,33^. The majority of these conclusions were drawn based on studies using rice cultivars with a shorter growth duration. In the present study, grain yield in YLY900 and YLY6 was not significantly correlated with either RRVO or CRVO (Fig. 4), which indicated that preheading reserves make a relatively minor contribution to grain yield of rice cultivars with a long grain-filling period. Instead, grain yield of YLY900 and YLY6 showed a significant positive correlation with the leaf SPAD value^28^. Interestingly, the Pearson correlation coefficients were increasingly higher and more strongly significant with progression of the developmental stage after heading (*r* = 0.2166^ ns^, *r* = 0.4219*, *r* = 0.5640** at the heading stage, 15 days after heading, and 30 days after heading, respectively; * *p* < 0.05, ** *p* < 0.01, ns = nonsignificant; Fig. 5). Consistent with previous observation that topdressing at the initial stage of flag-leaf extension resulted in the highest grain yield, the maximal significant correlation coefficient of 0.87 was observed at 15 days after heading (Fig. 6). These results suggested that rice cultivars with a long growth duration show novel characteristics, i.e., grain yield is strongly dependent on canopy photosynthetic CO_2_ uptake during the grain-filling stage, rather than organic carbon stored in the stem and sheath. Previous studies have estimated that during the grain-filling stage, canopy photosynthetic CO_2_ uptake may contribute 60–100% of the final grain yield of rice^30,31^ and more than 40% of canopy photosynthesis can be contributed by the flag leaves^34^. In the current study, on average, the contribution of canopy photosynthetic CO_2_ uptake to total grain yield formation in YLY900 and YLY6 was higher than 78% and 87%, respectively (Table 3), which was much higher than the percentages reported previously for other rice cultivars^35^.

## Conclusion

Compared with rice cultivars with a relatively shorter growth duration, topdressing must be applied at an appropriate stage of young panicle differentiation to realize the enhanced grain-yield potential of cultivars with a longer growth duration. Specifically, for the two cultivars tested in this study, topdressing at the initial stage of flag-leaf extension results in greater enhancement of grain yield, which is primarily contributed by the improved physiological activity of the flag leaves at the middle and advanced grain-filling stages. In contrast to earlier-maturing rice cultivars, in which remobilization of stored carbon in the stem and sheath contributes as much as 30–40% of the final grain yield, in YLY900 and YLY6 the photosynthetic CO_2_ uptake rate during the grain-filling stage contributed to more than 78%–87% of the final grain yield. The information provided in this study can be used to improve topdressing application for rice cultivars with a long growth duration.
